# Development of a Charge Adjustment Model for Cardiac Catheterization

**DOI:** 10.1007/s00246-014-0994-3

**Published:** 2014-08-12

**Authors:** Andrew Brennan, Kimberlee Gauvreau, Jean Connor, Cheryl O’Connell, Sthuthi David, Melvin Almodovar, James DiNardo, Puja Banka, John E. Mayer, Audrey C. Marshall, Lisa Bergersen

**Affiliations:** Department of Cardiology, Children’s Hospital Boston, Harvard Medical School, 300 Longwood Avenue, Boston, MA 02115 USA

**Keywords:** Resource utilization, RVU, Congenital heart disease, Catheterization, Outcomes

## Abstract

A methodology that would allow for comparison of charges across institutions has not been developed for catheterization in congenital heart disease. A single institution catheterization database with prospectively collected case characteristics was linked to hospital charges related and limited to an episode of care in the catheterization laboratory for fiscal years 2008–2010. Catheterization charge categories (CCC) were developed to group types of catheterization procedures using a combination of empiric data and expert consensus. A multivariable model with outcome charges was created using CCC and additional patient and procedural characteristics. In 3 fiscal years, 3,839 cases were available for analysis. Forty catheterization procedure types were categorized into 7 CCC yielding a grouper variable with an *R* ^2^ explanatory value of 72.6 %. In the final CCC, the largest proportion of cases was in CCC 2 (34 %), which included diagnostic cases without intervention. Biopsy cases were isolated in CCC 1 (12 %), and percutaneous pulmonary valve placement alone made up CCC 7 (2 %). The final model included CCC, number of interventions, and cardiac diagnosis (*R* ^2^ = 74.2 %). Additionally, current financial metrics such as APR-DRG severity of illness and case mix index demonstrated a lack of correlation with CCC. We have developed a catheterization procedure type financial grouper that accounts for the diverse case population encountered in catheterization for congenital heart disease. CCC and our multivariable model could be used to understand financial characteristics of a population at a single point in time, longitudinally, and to compare populations.

## Introduction

Congenital heart disease (CHD) accounts for a substantial amount of health care spending in the USA, with acute care expenditures estimated to be 6 billion dollars annually [11, 13, 14]. This large amount of healthcare expenditures is the result of the variable anatomical and physiological anomalies seen in these patients, the requirement for multiple hospitalizations for surgical and interventional therapies, and lifelong follow-up with multidisciplinary medical professionals [11]. Marelli et al. [8] in a study of the population in Quebec over the past several decades showed that mortality rates have decreased for patients with CHD. As CHD patients’ life expectancy increases so does the prevalence of CHD in the general population, leading to a greater need to understand the financial characteristics of this diverse population so that potential areas to reduce and standardize charges are identified.

We hypothesized that examining charges derived from Current Procedural Terminology (CPT) billing codes of catheterization procedures would provide a more accurate assessment of patient resource utilization than widely used current financial metrics such as the All Patient Refined Diagnosis Related Groups (APR-DRG) diagnostic classification system, severity of illness (SOI), and case mix index (CMI). Recent analysis has shown that the APR-DRG system inaccurately classifies patients admitted for congenital heart surgery, which may have an impact on the accuracy of outcome reporting and resource allocation planning [10]. We sought to create a procedure type financial grouper as well as consider other clinical or procedural characteristics known a priori to predict resource utilization using charges in CHD populations undergoing cardiac catheterization. Total procedure-related values based on CPT codes were chosen as the outcome for resource utilization to provide generalizability to other institutions as procedures are generally billed based on CPT codes.

## Methods

The study methods were reviewed and approved by the Institutional Review Board at Children’s Hospital Boston.

### Database Source

Our catheterization laboratory reporting and billing database include patient and procedural characteristics on all procedures performed at Children’s Hospital Boston. Hospital charges were matched to all catheterization cases in the clinical database. As total hospital charges for the majority of catheterization cases may reflect other aspects of care unrelated to the catheterization procedure, such as elective and surgical interventions during the same hospitalization, the outcome charges chosen by the group were total procedure-related values from the catheterization laboratory, comprising of procedure codes (CPT with associated charge), supplies, and recovery-related expenses. Recovery-related expenses were expenses accumulated during the patient’s recovery and observation time in the catheterization laboratory department and excluded any other aspects of in-hospital care, such as recovery time as an inpatient in the Intensive Care Unit or general floor. Cases were linked to an APR-DRG Version 20.0 value based on International Classification of Diseases, 9th revision, Clinical Modification (ICD-9-CM) procedure and diagnosis codes assigned in the reporting and billing database.

### Population

All cases classified as diagnostic, interventional, or biopsy only were identified in the catheterization database and included. Few common case types such as hybrids or cases recorded as pericardiocentesis, pleuracentesis, or fluoroscopy only were excluded. The fiscal years 2008, 2009, and 2010 comprised the cohort. In the data set, 3,978 cases met inclusion criteria of which 3,940 cases were matched in the hospital database and of these 3,883 had an associated charge recorded. Outliers with total charges less than $10,000 and greater than $100,000 were examined in detail. Based on this review, we excluded 16 cases with incomplete billing and a total charge less than $5,000 (*N* = 3,867) and excluded 28 with no procedure charge for a final cohort of 3,839 cases.

### Predictor Variables

We considered both patient and procedural characteristics as potential predictor variables of the outcome charges. Patient characteristics included the following: age, weight, diagnosis, genetic syndrome, non-cardiac problem, previous catheterization, previous catheterization within 30 days, previous surgery, previous surgery within 30 days, admit source, mechanical circulatory support including ECMO, continuous supportive intravenous (IV) medications including vasoactive medications and inotropes, intubation status, known vascular occlusion, and indicators of hemodynamic vulnerability. Procedural characteristics included the following: year of procedure, case type (diagnostic, interventional, or biopsy only), intervention type (balloon angioplasty, valvotomy, stent placement, stent redilation, device implant, or coil implant), number of interventions, and procedure type risk category as designated by Catheterization for Congenital Heart Disease Adjustment for Risk Method (CHARM) [2].

### Development of Procedure Types and Catheterization Charge Categories (CCC)

Since cardiac catheterization for CHD includes a wide variety of case types, a multidisciplinary panel comprising of the authors was established to develop procedure types (*n* = 35) with empiric analysis followed by expert consensus to group case types into categories with similar charges. Charges were summarized by mutually exclusive catheterization procedure types. To identify procedure types with potential modifying factors, procedure types were ranked from high to low distribution of charges within the procedure type designation. The procedure types were grouped into different combination of numbers of categories according to empiric similarity in order to minimize the variation within a group while maximizing discrimination between groups.

### Statistical Methods and Development of the Multivariable Model

Patient and procedural characteristics were summarized by median and interquartile range charges. The coefficient of determination (*R* ^2^) was calculated by univariate and multivariable linear regression models for the outcome charges. A multivariable model was built using stepwise forward regression for the outcome charges to normalize for skewed distributions. Starting with CCC, all of the patient and procedural characteristics were considered for inclusion in the multivariable model until no additional explanatory value could be found by adding a variable to the model. The proportion of cases in each of the 4 subclasses of APR-DRG SOI was summarized by CCC, and the weighted mean SOI was calculated by CCC. Geometric mean APR-DRG CMI was calculated by CCC.

## Results

### Patient Characteristics

Charges were summarized according to patient characteristics based on the final cohort of 3,839 cases in Table 1. The majority of the patients was between the ages of 1–18 (82 %) and had a diagnosis of complex CHD with two ventricles (39 %) or single ventricle physiology (19 %). For most cases, the patient had a previous catheterization (54 %) or a previous surgery (54 %). In univariate analysis although diagnosis alone explained some variation in charge (*R* ^2^ = 27.2 %), we noted that, as a population, the majority of patient characteristics that anecdotal experience suggests may be important in determining the intensity of resources needed to perform a case, such as age (*R* ^2^ = 2.9 %), admit source (*R* ^2^ = 0.2 %), intubation status (*R* ^2^ = 10.1 %), continuous supportive IV medications (*R* ^2^ = 0.1 %), and indicators of hemodynamic vulnerability (*R* ^2^ = 2.4 %), did not individually explain the variability in charges despite having a significant association with charge (*P* < 0.01).

**Table 1:** Summary of charges for catheterization by patient characteristics

	*N* (%)	Median ($)	Percentiles		*P* value	*R* ^2^
			25th	75th		
*Age*						
<1 month	227 (6)	26,843	17,136	33,839	<0.001	0.029
≥1 month, <1 year	659 (17)	32,351	20,538	44,909		
≥1 year, <5 years	913 (24)	33,540	19,617	45,392		
≥5 years, <18 years	1,340 (35)	22,846	15,782	37,942		
≥18 years	700 (18)	23,246	16,692	37,727		
*Weight (kg)*						
<4	321 (8)	28,759	17,646	35,660	<0.001	0.034
4–9	830 (22)	32,868	20,350	45,038		
10–19	815 (21)	33,787	19,818	45,769		
≥20	1,872 (49)	22,636	15,834	37,134		
*Diagnosis* ^a^						
No structural defects	150 (4)	16,676	11,949	23,039	<0.001	0.298
Heart transplant	743 (19)	15,126	13,335	20,632		
Isolated defects	635 (17)	31,337	21,636	36,807		
Pulmonary hypertension	107 (3)	16,784	10,086	21,724		
Complex two ventricle	1,491 (39)	34,235	21,006	48,927		
Single ventricle	711 (19)	37,014	24,004	50,281		
*Genetic syndrome* ^b^						
Yes	502 (13)	30,046	18,516	43,876	0.02	0.001
No	3,330 (87)	27,896	17,621	41,025		
*Non-cardiac problem* ^c^						
Yes	1,372 (36)	25,696	17,448	40,602	0.03	0.002
No	2,454 (64)	29,195	18,018	41,577		
*Previous catheterization*						
Yes	2,058 (54)	35,819	21,406	50,322	<0.001	0.151
No	1,781 (46)	20,605	15,069	31,832		
*Previous catheterization within 30 days*						
Yes	207 (5)	34,474	21,785	54,199	<0.001	0.008
No	3,632 (95)	27,573	17,676	40,899		
*Previous surgery*						
Yes	2,064 (54)	35,099	21,340	48,898	<0.001	0.143
No	1,775 (46)	20,549	14,854	32,239		
*Previous surgery within 30 days*						
Yes	229 (6)	29,451	18,708	43,172	0.11	0.001
No	3,610 (94)	27,955	17,757	41,173		
*Admit source*						
Elective	3,038 (79)	28,269	18,037	41,754	0.04	0.002
Non elective	801 (21)	28,046	17,103	40,068		
*Mechanical circulatory support* ^d^						
Yes	88 (2)	28,323	17,408	39,615	0.98	0.000
No	3,724 (97)	28,266	17,850	41,305		
*Continuous supportive IV medications* ^e^						
Yes	421 (11)	29,451	18,142	41,446	0.15	0.001
No	3,392 (88)	27,896	17,731	41,213		
*Intubation status* ^f^						
Spontaneous	1,393 (36)	20,216	15,094	32,173	<0.001	0.101
Prior to transfer	507 (13)	31,079	18,794	43,262		
Before case	1,886 (49)	33,565	20,476	47,682		
During case	40 (1)	37,359	18,727	53,733		
*Known vascular occlusion*						
Yes	507 (13)	30,548	18,660	48,133	0.002	0.003
No	3,332 (87)	27,914	17,716	40,716		
*Indicators of hemodynamic vulnerability*						
0	2,055 (54)	23,416	16,050	36,775	<0.001	0.024
1	1,018 (27)	31,416	19,123	45,050		
≥2	766 (20)	32,376	19,274	47,319		

^a^Not entered *N* = 2; ^b ^Not entered *N* = 7; ^c ^Not entered *N* = 13; ^d ^Not entered *N* = 27; ^e ^Not entered *N* = 26; ^f ^Not entered *N* = 13

### Procedural Characteristics

Charges were summarized according to procedural characteristics (Table 2). About 57 % of cases were interventional, with balloon angioplasty (27 %) the most frequent intervention performed. The majority of cases required two or fewer interventions (82 %). The increased median charge by year of catheterization procedure is mostly due to an increase in the number of percutaneous pulmonary valve placements performed over the three fiscal years 2008, 2009, and 2010. In univariate analysis, case type (*R* ^2^ = 54.4 %), number of interventions (*R* ^2^ = 49.8 %), and procedure type risk category (*R* ^2^ = 49 %) individually demonstrated a moderate relationship with the variability in charges. However, examining intervention type individually did not explain variability in charges although each had a significant association with charge (*P* < 0.01).

**Table 2:** Summary of charges for catheterization by procedural characteristics

	*N* (%)	Median ($)	Percentiles		*P* value	*R* ^2^
			25th	75th		
*Year of procedure*						
2008	1,238 (32)	25,952	16,584	38,219	<0.001	0.007
2009	1,363 (36)	28,854	18,454	42,839		
2010	1,238 (32)	30,001	18,211	41,734		
*Case type*						
Diagnostic	915 (24)	18,180	16,021	20,281	<0.001	0.544
Interventional	2,196 (57)	38,084	31,264	51,303		
Biopsy	728 (19)	15,059	13,253	20,468		
*Balloon angioplasty*						
Yes	1,021 (27)	43,895	33,830	57,876	<0.001	0.255
No	2,818 (73)	21,086	15,899	33,844		
*Valvotomy*						
Yes	242 (6)	34,947	31,457	40,980	<0.001	0.017
No	3,597 (94)	25,822	17,394	41,270		
*Stent placement*						
Yes	497 (13)	47,745	37,988	62,663	<0.001	0.154
No	3,342 (87)	23,396	16,868	36,682		
*Stent redilation*						
Yes	262 (7)	45,456	34,109	61,780	<0.001	0.061
No	3,577 (93)	25,698	17,353	39,725		
*Device implant*						
Yes	348 (9)	35,161	30,542	48,147	<0.001	0.028
No	3,491 (91)	25,545	17,183	40,731		
*Coil implant*						
Yes	445 (12)	47,199	37,130	61,359	<0.001	0.120
No	3,394 (88)	23,801	16,972	37,446		
*Number of interventions*						
None	937 (24)	18,206	15,984	20,315	<0.001	0.498
1	1,730 (45)	24,309	15,374	33,611		
2	493 (13)	40,980	34,491	48,976		
3	283 (7)	49,344	41,211	61,501		
4	184 (5)	55,190	47,080	67,397		
≥5	212 (6)	61,731	49,073	72,145		
*Procedure type risk category* ^a^						
1	1,403 (36)	16,764	13,785	20,419	<0.001	0.490
2	930 (24)	31,418	22,369	36,875		
3	883 (23)	39,251	30,848	53,035		
4	594 (16)	45,409	36,280	59,944		

^a^No category *N* = 29

### Procedure Types

For some procedure types, such as pulmonary artery angioplasty, discrimination improved when stratified by the number of interventions, while for other procedure types, such as pulmonary valve dilation, the number of interventions had less effect. Procedure types that led to improved discrimination were stratified into the number of interventions, leading to the final 40 procedure types based on expert clinical consensus and empiric analysis summarized in Table 3.

**Table 3:** Catheterization charge categories with the associated procedure types

	*N*	Median ($)	Percentiles	
			25th	75th
*Category 1*				
Heart biopsy after transplant	451	13,880	12,744	15,213
*Category 2*				
Heart biopsy and diagnostic catheterization	990	18,243	15,909	20,478
Heart biopsy and coronary angiography	263	20,860	16,800	22,847
Diagnostic catheterization including transseptal puncture	43	23,018	19,744	25,075
*Category 3*				
PDA device or coil closure	86	27,123	21,457	32,508
Atrial septum stent, dilation, or septostomy	62	28,961	22,155	40,712
PDA stent	4	28,439	27,304	31,322
Aorta dilation and/or stent (one intervention)	69	29,121	26,482	32,778
Aorta stent redilation	32	29,515	27,829	32,954
Pulmonary valvotomy (isolated intervention)	95	31,982	29,421	34,439
RVOT dilation and/or stent (no PA angioplasty or stent) (one intervention)	58	32,829	30,110	35,854
Proximal or distal right or left PA angioplasty and/or stent (excluding RVOT intervention) (one intervention)	116	32,868	29,380	37,600
*Category 4*				
Systemic vein dilation or stent	72	33,007	25,069	42,564
ASD or PFO closure (isolated intervention)	164	33,696	30,790	36,754
Aortic valvotomy (isolated intervention)	79	35,453	32,849	38,314
Coil/device systemic collateral (one intervention)	62	35,864	32,512	39,693
Systemic pulmonary shunt dilation or stent	16	37,746	28,365	47,257
Fenestration device closure (isolated intervention)	21	36,197	34,296	37,772
Coronary fistula closure	6	38,282	35,819	39,596
Systemic artery angioplasty and/or stent (not aorta)	22	40,210	27,898	46,423
RVOT dilation and/or stent and proximal or distal right or left PA angioplasty and/or stent (two interventions)	34	37,936	35,168	45,702
Fontan baffle dilation	21	36,643	32,903	43,554
*Category 5*				
RVOT dilation and/or stent (no PA angioplasty or stent) (plus at least one additional intervention)	37	43,702	34,967	48,210
Any pulmonary vein dilation and/or stent intervention	105	41,032	35,017	48,844
Aortic valvotomy (plus at least one additional intervention)	7	41,530	29,957	57,017
Proximal or distal right or left PA angioplasty and/or stent (excluding RVOT intervention) (two interventions)	124	41,870	36,338	48,759
Aorta dilation and/or stent (plus at least one additional intervention)	53	42,822	37,652	48,386
Systemic collateral coil or device closure (plus at least one additional intervention)	122	42,859	37,130	54,854
Pulmonary valvotomy (plus at least one additional intervention)	7	46,151	39,433	52,986
Systemic venous collateral coil or device closure	24	40,142	36,733	52,169
Mitral valvotomy	24	43,129	40,464	52,788
RVOT dilation and/or stent and proximal or distal right or left PA angioplasty and/or stent (three interventions)	32	49,014	40,553	56,045
VSD device placement	15	44,904	42,908	53,466
Systemic pulmonary collateral dilation or stent	4	49,382	39,240	59,163
*Category 6*				
Proximal or distal right or left PA angioplasty and/or stent (excluding RVOT intervention) (three interventions)	109	50,821	43,451	60,418
Fenestration device closure (plus at least one additional intervention)	39	53,480	48,161	63,497
ASD or PFO closure (plus at least one additional intervention)	9	57,114	43,846	70,837
RVOT dilation and/or stent and proximal or distal right or left PA angioplasty and/or stent (four or more interventions)	36	62,001	55,281	69,004
Proximal or distal right or left PA angioplasty and/or stent (excluding RVOT intervention) (four or more interventions)	198	60,347	50,675	70,345
*Category 7*				
Percutaneous pulmonary valve placement	89	83,095	75,848	90,434

*PDA* patent ductus arteriosus, *RVOT* right ventricular outflow tract, *PA* pulmonary artery, *ASD* atrial septal defect, *PFO* patent foramen ovale, *VSD* ventricular septal defect

### Catheterization Charge Categories

With the procedure types defined, the multidisciplinary panel looked for a categorization number that had face validity, with the final CCC containing 7 categories as summarized in Table 3. Although similar in charges to diagnostic cases, biopsy cases were forced as a separate group in the CCC for better potential future generalizability of the final model when applying to institutions that perform diagnostic, but few if any biopsy cases. Percutaneous pulmonary valve placement was forced into a separate category for similar reasons. Additionally, the total procedure-related charges for percutaneous pulmonary valve placement, for which a high percentage is associated with the cost of the valve device, were significantly higher than those associated with any other procedure type.

**Fig. 1:**
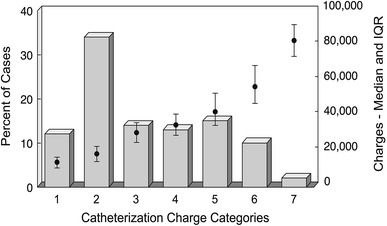
Distribution of cases and associated charges by CCC. The distribution of catheterization cases by CCC is represented by the columns, and the calculated median and interquartile range charge for each CCC is represented by the scatter plot

The distribution of cases by CCC and the median and interquartile range of associated charges by CCC are shown in Fig. 1. The largest proportion of cases were ranked in CCC 2 (34 %), containing mostly diagnostic cases without intervention. A summary of univariate comparison of catheterization charges by CCC is detailed in Table 4, with a statistically significant *p* value for all categories (*P* < 0.01). The univariate linear regression model for the outcome charges demonstrated a moderately strong relationship (*R* ^2^ = 72.6 %) between variability in charges by CCC.

**Table 4:** Catheterization charge category distribution

	*N* (%)	Median ($)	Percentiles		*P* value	*R* ^2^
			25th	75th		
*Catheterization charge category*						
1	451 (12)	13,880	12,744	15,213	<0.001	0.726
2	1,296 (34)	18,786	16,170	21,628		
3	522 (14)	30,869	26,966	34,811		
4	497 (13)	35,222	31,368	39,925		
5	554 (15)	42,773	36,884	51,695		
6	391 (10)	56,926	48,966	67,523		
7	89 (2)	83,095	75,848	90,434		

### Multivariable Model

A multivariable linear regression model was developed for the outcome, procedure-related values, starting with CCC and including patient and procedural characteristics from the univariate model. The addition of number of interventions (*R* ^2^ = 73.7 %) and number of interventions and diagnosis (*R* ^2^ = 74.2 %) minimally improved explanation of the variability in charges, while all other characteristics added such as case type, age, and admit source did not result in a significant increase in explanatory value.

### Catheterization Charge Category and APR-DRG Relationship

**Fig. 2:**
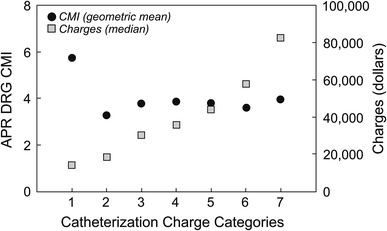
APR-DRG CMI and associated charges. Catheterization cases with an associated APR-DRG value were linked to CMI subclass and grouped into corresponding CCC to allow for median charge as well as geometric mean CMI to be calculated by CCC

We were able to link 76 % (*N* = 2,919) of cases performed as inpatients to an APR-DRG SOI and CMI value. These values were examined to determine whether they correlated with CCC, and the final results are summarized in Table 5 and Fig. 2. The total number of cases linked to an APR-DRG value was grouped into the corresponding CCC, and the median charge for each CCC was calculated. The weighted mean SOI for CCC 1 (3.3) when compared to CCC 7 (2.4) demonstrated no relationship between CCC and SOI subclasses. Furthermore, the percentage of all cases classified as SOI subclass 4, the highest SOI subclass, was 44 % in CCC 1 compared to 2 % in CCC 7. Similarly, CMI proved to have little association with CCC. Instead of a direct relationship between geometric mean CMI and CCC, CCC 1 (5.8) had a higher geometric mean CMI than any other CCC, including CCC 7 (4.1) (Fig. 2).

**Table 5:** APR-DRG severity of illness and case mix index

Catheterization charge category	Cases	Median	APR-DRG v20 SOI					APR-DRG v20 CMI
			1	2	3	4	Weighted	Geometric
	*N* (%)	Charge ($)	*N* (%)	*N* (%)	*N* (%)	*N* (%)	Mean SOI	Mean CMI
1	119 (4)	13,989	2 (2)	18 (15)	47 (40)	52 (44)	3.3	5.8
2	863 (30)	18,404	78 (9)	278 (32)	270 (31)	237 (27)	2.8	3.4
3	434 (15)	30,301	53 (12)	169 (39)	125 (29)	87 (20)	2.6	3.8
4	456 (16)	35,838	136 (30)	130 (29)	116 (25)	74 (16)	2.3	3.9
5	532 (19)	43,847	16 (3)	208 (39)	207 (39)	101 (19)	2.7	3.8
6	382 (13)	57,692	10 (3)	160 (42)	160 (42)	52 (14)	2.7	3.7
7	85 (3)	82,746	3 (4)	44 (52)	36 (42)	2 (2)	2.4	4.1

*CMI* case mix index, *SOI* severity of illness

## Discussion

To more accurately account for the variability of charges in CHD patients undergoing a catheterization procedure, our multidisciplinary group developed a metric based on catheterization charges from the reporting and billing database at Children’s Hospital Boston for fiscal years 2008–2010. The outcome chosen to account for resource utilization was total procedure-related values, which comprised of procedure codes (CPT with associated charge), supplies, and recovery-related expenses. Procedure-related values were chosen in order to exclude charges and aspects of in-hospital care during the episode of care unrelated to the catheterization laboratory. Catheterization cases were defined into 40 procedure types by empiric analysis followed by expert consensus. The procedure types were further grouped into 7 CCC stratified by charge outcomes. The CCC demonstrated a moderately strong explanation of the variability in outcome charges (*R* ^2^ = 72.6 %). Factors typically associated with resource utilization such as number of interventions (*R* ^2^ = 73.7 %) and number of interventions and diagnosis (*R* ^2^ = 74.2 %) slightly improved discrimination when added to the CCC.

The APR-DRG diagnostic classification system, a widely used financial metric that evaluates resource intensity and outcomes, is coded based on hospital discharge data and is therefore inadequate in serving as a prospective predictor of resource utilization. As APR-DRG is based on the accumulation of a patient’s characteristics including all cardiac and non-cardiac diagnoses, comorbidities, and past procedures, this metric may be less adequate in measuring patient resource utilization than CCC, which is based on procedure-related charges and modifiers derived from a priori patient and procedural characteristics reflecting the patient’s health status at the time of the catheterization. In our analysis, geometric mean CMI was highest in CCC 1, comprised of heart biopsy after transplant, although this was the procedure type with the lowest associated median charge. This finding may be due to this patient population’s significant past cardiac diagnoses and procedures rather than the resource utilization required at the time of a heart biopsy catheterization. Neither SOI or CMI demonstrated a relationship with CCC and as SOI and CMI are intended to capture the financial complexity of patients we expected that these metrics would correlate with CCC as we have shown CCC measures the variation in charges and resource utilization for catheterization procedures reasonably well.

The majority of Medicare and non-Medicare payers report current use of a system of physician reimbursement based on the Medicare Resource-Based Relative Value Scale (RBRVS), with 77 % of private plans based on this payment system according to an American Medical Association (AMA) survey in 2006 [1, 4, 6]. Physician payment through the RRBVS system is determined by total relative value units (RVUs), which are based on physician work, professional expenses, and professional liability insurance, and are assigned to procedures identified by the AMA’s CPT codes [1, 6]. In the field of congenital heart surgery, CPT codes and subsequently work RVUs were assigned to 81 congenital heart operations in 1993–1994 as they previously did not exist for these procedures. Jenkins et al. [7] validated that the work RVU scale for congenital heart operations correlated reasonably with measuring physician work and resource consumption.

The catheterization procedure types and CCC developed by our group are derived from CPT-associated billing codes similar to the CPT-based RVU system. Our multidisciplinary panel believes that our developed metric presents an easily generalizable tool to assess catheterization charges among institutions as many institutions bill for procedures based on CPT codes. Catheterization cases can be linked from reporting and billing databases to one of the 40 developed procedure types and the corresponding CCC to calculate the median charge of each CCC at various institutions. This is especially pertinent as the APR-DRG system has been found to inaccurately classify patients admitted for congenital heart surgery, which can present inaccurate outcome reporting in center comparison [10].

Recently, the CPT-based RVU system’s ability to accurately reflect physician work has been examined and been found inadequate in several surgical fields [9, 12]. One concern is whether work RVUs accurately measure physician work in pediatric procedures as the RBRVS was developed to estimate work on standard adult Medicare patients [4]. The CPT-based RVU system assigned to pediatric CHD catheterization procedures has also been found to be inadequate in reflecting required physician work in a study by Bergersen et al. [3]. This study concluded that many of the pediatric catheterization CPT codes did not exist or were deduced from procedures performed in adults [3]. Thus, this system does not adjust for the variable age range or the variable complexity within and between catheterization procedures [3]. For instance within diagnostic catheterizations, CPT codes (93530–93533), a routine angiogram of an otherwise healthy patient is reimbursed at a similar rate as an angiogram for an inpatient infant following surgery although the resources, length of procedure, and number of full-time equivalent (FTE) ancillary personnel required differ significantly. Likewise, the CPT code (93580) for an isolated atrial septal defect (ASD) or patent foramen ovale (PFO) closure, which is typically a short intervention with low adverse event rates, has a much higher RVU than diagnostic catheterizations and is on par with more difficult procedures such as pulmonary vein dilations [3].

The inadequacies inherent in deriving physician work and resource utilization from RVU assigned to CPT codes means our developed metric has similar shortcomings as those described. This led our group to examine potential modifiers based on a priori patient and procedural characteristics that may further strengthen the metric and account for these inadequacies. Stratification via expert consensus by the number of interventions of the catheterization procedure types and the addition of the number of interventions and diagnosis to CCC resulted in a metric that is a moderately strong predictor of charges. However, our metric warrants reevaluation, especially in its ability to measure resource intensity and physician work during a catheterization. In order to add explanatory value, additional characteristics may need to be measured and accounted for such as complexity of care requiring different levels of resources from a staffing perspective. Duration of procedure is a potential characteristic that has been indicated as an area that the current CPT-based RVU system does not reimburse appropriately [9, 12].

The development of the procedure types and CCC was based on catheterization data from a single institution, and after reevaluation, further validation will be required to demonstrate our metric is generalizable to cases at different institutions. The value of absolute hospital charges assigned to procedure types may vary between institutions due to the valuation of an interventional procedure CPT code, hospital location, and local billing patterns; however, relative hospital charges by catheterization procedure type and CCC should be generalizable. Attempting comparative analyses in administrative databases such as the Pediatric Health Information System (PHIS), an inpatient dataset, was determined to not be possible because most catheterization cases are considered outpatient procedures. Therefore, exploration of our hospital finance data revealed that less than 50 % of catheterization cases are captured in PHIS.

The developed procedure type financial grouper shows that it is feasible to create such a predictive model for catheterization. Studies examining the variation in costs among institutions and charge methodologies associated with CHD patients in pediatric cardiology have mostly focused on surgical management up to this point [5, 7, 11, 15]. However, catheterization procedures for CHD patients warrant a separate methodology to compare charges, yet no methodology nor billing standards currently exist. The inadequacies with the current RBRVS system justifies a reevaluation of the current billing system and an expanded set of CPT codes to better capture the variability in age, complexity, and the procedures types performed for CHD patients undergoing catheterization.

## Conclusion

The results of this study demonstrate that the development of a procedure type financial grouper for CHD catheterization case types is feasible. The metric developed by our group, CCC, is a more accurate tool in assessing patient resource utilization than current financial metrics, such as APR-DRG, case mix index, and severity of illness as both of these financial metrics did not correlate with CCC. Although CCC is a moderately strong predictor of the variability in catheterization procedure type charges, further work is needed to identify additional patient and procedural characteristics that influence resource utilization and physician work as they may strengthen the metric. In the interim, as CCC relies on defining procedures via CPT codes and catheterization procedures at institutions are generally billed based on CPT codes, this developed metric provides an easily generalizable tool to examine charges at a single point in time, longitudinally, and across institutions to inform negotiation of prices with health care reformers.
